# Leader–Following Fault-Tolerant Consensus Control for Multi-Agent Systems Based on Observers

**DOI:** 10.3390/s26103153

**Published:** 2026-05-16

**Authors:** Tengzi Liu, Fanglai Zhu, Haichuan Xu

**Affiliations:** 1 Shanghai Research Institute for Intelligent Autonomous Systems, Tongji University, Shanghai 200092, China; 2411886@tongji.edu.cn (T.L.); xuhaichuan@tongji.edu.cn (H.X.); 2 School of Electronics and Information Engineering, Tongji University, Shanghai 201804, China

**Keywords:** distributed observer, multi-agent systems, unknown input observer, fault reconstruction, DO-based distributed control protocol

## Abstract

In this paper, for leader–follower structure multi-agent systems (MASs), a new fault-tolerant consensus control mechanism which is called the distributed information estimation and centralized control scheme is developed. To begin with, for each follower agent, an unknown input observer (UIO) is designed to obtain the asymptotic convergence state estimation. Then, a fault reconstruction (FR) method is proposed through constructing an interval observer by sensor measurement output. Most importantly, using the leader’s state estimation provided by the local observer, a distributed observer (DO) is designed so that each follower can obtain the leader’s state estimation. Subsequently, for each follower agent, by using its own state estimation and FR, and the leader’s state estimation offered by the DO, a centralized controller is designed. In this way, a DO-based distributed fault-tolerant control protocol is developed, in which the distributed feature is majorly reflected by the DO construction, resulting in the controller being formulated in a centralized way. In addition, under the DO-based distributed fault-tolerant control protocol, MAS consensus can be reached. Finally, two simulation examples are given to show the effectiveness of the proposed methods.

## 1. Introduction

Distributed observer (DO) is typically composed of multiple local observers, providing a method for each agent to estimate the system’s state solely based on its own measurements and information received from its neighbors. The earliest systematic work on DO was proposed in 2007 [1]. Then, it is used for real-time monitoring, collecting, and analyzing of state information of various nodes in the system, in order to achieve global or local state estimation. Distributed cooperative control plays an extremely important role in MASs, such as energy management [2], unmanned aerial vehicle (UAV) formation [3,4], satellite formation [5], intelligent transportation systems [6], and so on. The leader–following consensus problems have been investigated for years, and many significant results can be found in the literature [7,8,9,10,11,12,13]. For example, Ref. [8] addresses the leader–following reliable consensus control problem, and proposes a non-fragile distributed consensus control protocol which can achieve MAS consensus even in the presence of stochastic parameter changes, actuator faults, and controller gain perturbations. Wang et al. [9] tackle the leader–follower consensus problem in MASs by formulating it as a particle swarm optimization problem. Zhang et al. [14] investigate disturbed event-triggered fixed-time bipartite consensus for nonlinear MASs with and without a leader over signed networks. In other aspects, leader–following consensus control involves an event-triggering control problem [15], switching topology problem [16], Denial-of-Service (DoS) attack problem [17], unknown input problem [18], containment control problem [19], and so on.

Under the leader–follower structure, the state information of the leader actually acts as one of the most crucial pieces of information that characterizes the status of MASs. For leader–following consensus problems, letting the followers obtain the leader’s state information directly or indirectly via communication networks will surely bring many benefits such that the problems can be solved much more conveniently. Ref. [20] for the first time explicitly introduces DOs into MASs to solve the problem of leader state estimation. Ref. [21] introduces an auxiliary system, transforms the original problem into an augmented system that includes the dynamics of agents and the leader, and adopts a discounted performance criterion along with the RARE (Robust Algebraic Riccati Equation) to rigorously solve the robust output consensus problem. Wang et al. [22] proposes a learning-based fully distributed observer that enables each agent to simultaneously learn the unknown dynamics and states of a nonlinear leader, and then designs an adaptive distributed control law to achieve leader–following consensus in heterogeneous Euler–Lagrange MASs. Recently, Bi et al. [23] proposed a novel adaptive distributed observer that estimates both the leader’s system matrix and state under unbounded distributed communication delays, and designed a distributed controller to achieve leader–following consensus.

It is widely recognized that actuators are susceptible to failures, as they are required to operate continuously and under varying conditions over extended periods. Consequently, the study of actuator fault-tolerant control has become critically important for MASs, as it enhances system reliability and ensures stable performance even in the presence of component malfunctions. A distributed adaptive leader–follower fault-tolerant controller is proposed for nonlinear MASs with actuator faults and delays [24]. Distributed bipartite adaptive event-triggered fault-tolerant leader–following consensus is investigated for MASs with unknown actuator faults [25].  In addition, the UIO is introduced to estimate the states of systems or even unknown inputs (UIs). Notably, in the context of actuator fault-tolerant control for MASs, actuator faults can be effectively modeled as a class of UIs. This equivalence allows the UIO to serve as a powerful tool not only for state estimation but also for real-time fault reconstruction, thereby providing a solid foundation for active fault-tolerant control strategies. And some results of handling UIs have been obtained [26,27,28,29]. For example, in [27], a distributed UIO (DUIO) is designed using a distributed interval observer for multi-sensor systems to estimate the states of the target system. UIO techniques are employed to address the challenges posed by unknown disturbances in MASs [28]. In [29], by combining a reduced-order observer with an interval observer, this paper presents a framework for fault reconstruction (FR) and fault-tolerant control that addresses both actuator and sensor faults concurrently. A clear and intuitive contrast with Fault-Tolerant Control and Observer Design is shown in Table 1.

In this article, for the MAS fault-tolerate consensus problem, a DO-based control protocol constructing scheme is developed such that the distributed feature is majorly reflected in DO construction. The main contributions of this article are as follows:

(1) Different from the work in [22,30,31], the followers considered in the present paper are subject to unknown disturbances and their states are unmeasurable. Thereby, a local UIO is designed for each follower to estimate the system state asymptotically. Moreover, through an interval observer, which is designed by sensor measurement output, an algebraic correlation linking the fault signal to the state estimation error is established. And then, an FR scheme, which can estimate the actual fault asymptotically, is developed. The proposed FR scheme can provide an asymptotically convergent estimate of the actual fault signal. This ensures that the reconstructed fault accurately approaches the true fault over time, thereby providing a highly precise baseline for generating a system compensation control scheme.
(2) By using the state estimation of the leader which is offered by a local Luenberger-like observer and the information of the neighbors, a DO is constructed at each follower agent. Unlike fully distributed control protocols [32,33] that suffer from heavy communication burdens and complex gain-tuning coupled with network topologies, this structural separation allows the controller to be formulated in a simple centralized way, significantly reducing inter-agent communication overhead. The proposed DO is able to estimate the leader’s state asymptotically. Therefore, through the DO, each follower can access the leader’s state information. Consequently, a DO-based controller for each follower can be developed to fulfill the MAS consensus in a centralized way.
(3) A DO-based fault-tolerant control protocol mechanism is developed through the combination of a simple feedback controller based on the state and FR. The proposed DO-based fault-tolerant control protocol can be viewed as a distributed control protocol, while the distributed feature is majorly reflected by the DO rather than the controller. In fact, because of the DO together with the local UIO, the controller becomes a simple state and FR feedback controller. More importantly, distinct from prior methods that only prove deterministic convergence, our framework mathematically establishes rigorous $H_{\infty }$ performance guarantees via Linear Matrix Inequalities (LMIs) shown in Theorem 1. In this way, the asymptotic convergence MAS consensus is reached.

The rest of this article is organized as follows. In Section 2, some preliminaries and the system description are presented. In Section 3, a UIO is designed for each follower to obtain the state estimation and the FR. In Section 4, a DO is designed such that each follower is able to access the leader’s state information, and then, a DO-based control protocol scheme is proposed. In Section 5, two simulation examples are applied. Section 6 gives the conclusions.

**Table 1:** Standardized methods summary of fault-tolerant control and observer design.

Paper	System	Fault Types	Observer	Control Architecture	Stability Guarantees
[34]	Nonlinear	Actuator	FO	Partially centralized	Finite-time
[35]	Nonlinear	∖	∖	Fully distributed	Finite-time
[36]	Linear	∖	ESO	Partially centralized	Exponential
[29]	Linear	Actuator, Sensor	Luenberger/Interval	Partially centralized	Asymptotic
Proposed	Linear	Actuator	UIO, Interval, DO	Partially centralized	Asymptotic

## 2. Preliminaries and System Description

In this section, the basic graph theory is introduced, and some notations are introduced. System description and lemmas are presented.

### 2.1. Graph Theory

The communication between agents can be represented by a topology graph G=(V,E,A), where V={1,…,N} represents the set of nodes, and E⊆V×V is the edge set. $A=\left[a_{ij}\right]\in R^{N\times N}$ denotes the adjacent matrix of G. (i,j)∈E means that agent *i* can receive the information from agent *j*, and agent *j* is a neighbor node of agent *i*. If (i,j)∈E, we set $a_{ij}>0$; otherwise, $a_{ij}=0$. The Laplacian matrix $L=\left[l_{ij}\right]\in R^{N\times N}$ is defined as $l_{ij}=-a_{ij}$ for i≠j and $l_{ii}=\sum_{j=1}^{N}a_{ij}\left(j\in N=\left\{1,\ldots ,N\right\}\right)$. Obviously, if we always assume that $a_{ii}=0$ and define $B=\underset{i\in N}{\mathrm{diag}}\left(l_{ii}\right)$, then we have L=B−A. Agent 0 is the leader agent. For follower agent *i*, if it can obtain the state information of the leader then $a_{i0}=1$; otherwise, $a_{i0}=0$. Define matrix H=L+D, where $D=\underset{i\in N}{\mathrm{diag}}\left(a_{i0}\right)$.

### 2.2. Notations

The Kronecker product is represented by the sign ⊗. ∥·∥ stands for the Euclidean norm of a vector. $I_{N}$ and $1_{N}$ represent an N×N identity matrix and N×1 vector with all entries being one, respectively. Define a matrix $S=\left[s_{ij}\right]\in R^{n\times m}$; let $S^{+}=\left[s_{ij}^{+}\right]$ with $s_{ij}^{+}=\max\left\{0,\;s_{ij}\right\}$ and $\;S^{-}=\left[s_{ij}^{-}\right]$ with $s_{ij}^{-}=\max\left\{-s_{ij},\;0\right\}$; then obviously, we have $S=S^{+}-S^{-}$, $\left|S\right|=S^{+}+S^{-}$. $S^{†}$ is generalized inverse of S satisfying $SS^{†}S=S$. Define another matrix $R=\left[r_{ij}\right]\in R^{n\times m}$; then S≤R if and only if $s_{ij}\leq r_{ij}\left(i=1,\ldots ,n;j=1,\ldots ,m\right)$.

### 2.3. System Description

Consider MASs consisting of N+1 agents: one leader and *N* followers. Suppose we have the follower system

(1) $$\left\{\begin{array}{c} {\dot{x}}_{i}=Ax_{i}+B\left(u_{f,i}+\omega_{i}\right)\\ y_{i}=Cx_{i},\;i=1,\ldots ,N \end{array}\right.$$

where A,B and *C* are known constant matrices with appropriate dimensions. $x_{i}\in R^{n},$$\;y_{i}\in R^{p}$ and $\omega_{i}\in R^{m}$ are respectively the system state, sensor measurement output and external disturbance of the *i*th agent. $\;u_{f,i}\in R^{m}$ represents the actuation signal, formulated as $u_{f,i}=\eta_{i}u_{i}+f_{a,i}$, where $\eta_{i}\in R$ and $f_{a,i}\in R^{m}$ denote the efficiency factor and bias signal of actuator faults, respectively. The actuator operates nominally provided that $\eta_{i}=1$ and $f_{a,i}=0$; conversely, any other values signify an actuation failure. Incorporating these fault parameters, the initial plant (1) is mathematically described by

(2) $$\left\{\begin{array}{c} {\dot{x}}_{i}=Ax_{i}+B\left(u_{i}+d_{i}\right)\\ y_{i}=Cx_{i},\;i=1,\ldots ,N \end{array}\right.$$

where $u_{i}$ stands for actuator control input, and $d_{i}=\left(\eta_{i}-1\right)u_{i}+f_{a,i}+\omega_{i}$ can be conceptually viewed as a multiple unknown input (MUI) within the dynamics in (2). The leader agent is described by

(3) $$\left\{\begin{array}{c} {\dot{x}}_{0}=Ax_{0}+Bu_{0}\\ y_{0}=Cx_{0} \end{array}\right.$$

where $x_{0}\in R^{n},\;u_{0}\in R^{m}$ and $y_{0}\in R^{p}$ are respectively the state, control input, and sensor measurement output of the leader.

The leader–follower consensus condition is defined as follows.

**Definition** **1.** *Consider MASs (2) and (3), if*

$$\lim_{t\to \infty }∥x_{i}\left(t\right)-x_{0}\left(t\right)∥=0,\;i=1,2,\ldots ,N.$$

*Then, it is said that the MASs (2) and (3) achieve consensus.*

**Assumption** **A1** ([37])**.** *System (2) is a minimum phase system; that is, the following rank condition*

$$\mathrm{rank}\left[\begin{array}{cc} sI_{n}-A & B\\ C & 0 \end{array}\right]=n+m$$

*is satisfied for all complex s with* Re(s)≥0.

**Assumption** **A2** ([37])**.** *The observer conditions rank(CB)=rank(B)=m holds.*

**Assumption** **A3.** *The pair (A,B) is stabilizable.*

**Assumption** **A4.** *The signed directed graph G associated follower is strongly connected.*

**Assumption** **A5** ([38])**.** *The external disturbance $\omega_{i}\left(t\right)$ is bounded with ${\underline{\omega }}_{i}\leq \omega_{i}\left(t\right)\leq {\bar{\omega }}_{i}$, where ${\underline{\omega }}_{i}$ and ${\bar{\omega }}_{i}$ are known constant vectors. The initial state $x_{i}\left(0\right)$ is unknown but bounded with ${\underline{x}}_{i,0}\leq x_{i}\left(0\right)\leq {\bar{x}}_{i,0}$, while ${\underline{x}}_{i,0}$ and ${\bar{x}}_{i,0}$ are constant known vectors. The actuator control input $u_{i}$, efficiency parameter $\eta_{i}$, and bias signal $f_{a,i}$ are bounded with ${\underline{u}}_{i}\leq u_{i}\left(t\right)\leq {\bar{u}}_{i}$, ${\underline{\eta }}_{i}\leq \eta_{i}\left(t\right)\leq {\bar{\eta }}_{i}$, ${\underline{f}}_{a,i}\leq f_{a,i}\left(t\right)\leq {\bar{f}}_{a,i}$ respectively, where ${\underline{u}}_{i}$, ${\bar{u}}_{i}$, ${\underline{f}}_{a,i}$ and ${\bar{f}}_{a,i}$ are known constant vectors, and ${\underline{\eta }}_{i}$ and ${\bar{\eta }}_{i}$ are known constant scalars.*

Under Assumption 5, we have ${\underline{d}}_{i}\leq d_{i}\left(t\right)\leq {\bar{d}}_{i}$, where ${\underline{d}}_{i}={\underline{\eta }}_{i}{\underline{u}}_{i}-{\bar{u}}_{i}+{\underline{f}}_{a,i}+{\underline{\omega }}_{i}$ and ${\bar{d}}_{i}={\bar{\eta }}_{i}{\bar{u}}_{i}-{\underline{u}}_{i}+{\bar{f}}_{a,i}+{\bar{\omega }}_{i}$. In practice, physical parameter variations and unmodeled dynamics are inevitable. This paper lumps these internal uncertainties and external disturbances into the bounded disturbance term $\omega_{i}\left(t\right)$. Under Assumption 5, the proposed DO-based protocol actively compensates for these combined uncertainties.

**Remark** **1.** *Assumptions 1 and 2 are the so-called minimal phase condition and observer matching condition, respectively. The former guarantees the convergence property of the state observer error system, while the latter means that the faults can be measured through the system outputs.*

**Remark** **2.** *The proposed strategy provides a unified solution for the diverse fault conditions outlined in Table 2 by constructing an augmented MUI: $d_{i}=\left(\eta_{i}-1\right)u_{i}+f_{a,i}+\omega_{i}$. The primary methodology relies on synthesizing an MUIR to asymptotically estimate the actual $d_{i}$, which is subsequently integrated into the compensation controller to guarantee the asymptotic stability of the closed-loop system. It is worth emphasizing that, in the case of $\eta_{i}\neq 1$, the constructed MUI explicitly contains the control input $u_{i}$. Due to coupling between $d_{i}$ and $u_{i}$, successfully executing the aforementioned two-step compensation strategy poses a significant challenge.*

## 3. Local Observer Design with FR

In this section, local observers are designed to obtain the state estimations of both the leader and followers. In addition, an FR design method is proposed.

### 3.1. Local Observer for Leader

A Luenberger observer is designed for (3)

(4) $${\dot{\hat{x}}}_{0}=A{\hat{x}}_{0}+Bu_{0}+L\left(y_{0}-C{\hat{x}}_{0}\right)$$

to obtain the state estimation of the leader. The error dynamic system between (3) and (4) is

(5) $${\dot{\tilde{x}}}_{0}=\left(A-LC\right){\tilde{x}}_{0}$$

where ${\tilde{x}}_{0}=x_{0}-{\hat{x}}_{0}$. In addition, we design the observer-based state feedback controller for the leader as

(6) $$u_{0}=K_{0}{\hat{x}}_{0}$$

Consequently, the closed-loop dynamics under the observer-based state feedback can be expressed as:

(7) $${\dot{x}}_{0}=\left(A+BK_{0}\right)x_{0}-BK_{0}{\tilde{x}}_{0}$$

### 3.2. UIO and FR Designs for the Followers

By the observer matching condition in *Assumption 2*, ${\left(CB\right)}^{†}={\left[{\left(CB\right)}^{T}CB\right]}^{-1}{\left(CB\right)}^{T}$ exists. Then, we have $(I_{n}+SC)B=0$ where $S=-B{\left(CB\right)}^{†}+\Gamma \left(I_{p}-CB{\left(CB\right)}^{†}\right)$ and $\Gamma \in R^{n\times p}$ is an arbitrary matrix. Therefore, multiplying the matrix $\left(I_{n}+SC\right)$ by the state equation of (2) from the left direction gives ${\dot{x}}_{i}=\left(I_{n}+SC\right)Ax_{i}-S{\dot{y}}_{i}$. Furthermore, by a state shift transformation of $\zeta_{i}=x_{i}+Sy_{i}$, System (2) is transformed into

(8) $$\left\{\begin{array}{c} {\dot{\zeta }}_{i}=\overset{⌢}{A}\zeta_{i}-\overset{⌢}{A}Sy_{i}\\ {\overset{⌢}{y}}_{i}=C\zeta_{i} \end{array}\right.$$

where $\overset{⌢}{A}=\left(I_{n}+SC\right)A$ and ${\overset{⌢}{y}}_{i}=\left(I_{p}+CS\right)y_{i}$.

**Lemma** **1.** *Under Assumption 1, the pair $\left(\overset{⌢}{A},C\right)$ is detectable with $\overset{⌢}{A}=\left(I_{n}-B{\left(CB\right)}^{†}C\right)A$.*

**Proof:** Refer to Appendix A.

Now, design a UIO for (8) as follows:

(9) $$\left\{\begin{array}{c} {\dot{\hat{\zeta }}}_{i}=\overset{⌢}{A}{\hat{\zeta }}_{i}-\overset{⌢}{A}Sy_{i}+\overset{⌢}{L}\left({\overset{⌢}{y}}_{i}-C{\hat{\zeta }}_{i}\right)\\ {\hat{x}}_{i}={\hat{\zeta }}_{i}-Sy_{i} \end{array}\right.$$

Then, the UIO error dynamic system can be obtained by subtracting (9) from (8):

(10) $${\dot{\tilde{\zeta }}}_{i}=\left(\overset{⌢}{A}-\overset{⌢}{L}C\right){\tilde{\zeta }}_{i}$$

where $\tilde{\zeta }=\zeta_{i}-{\hat{\zeta }}_{i}$. Therefore, (9) is a UIO of (8) or (2) by designing the observer gain matrix $\overset{⌢}{L}$ such that all the eigenvalues of $\overset{⌢}{A}-\overset{⌢}{L}C$ are with negative real parts, and the existence of the gain matrix $\overset{⌢}{L}$ can be guaranteed by Lemma 1. That is ${\hat{\zeta }}_{i}$ and ${\hat{x}}_{i}$ are the asymptotic convergent estimations of $\zeta_{i}$ and $x_{i}$, respectively. In addition, denoting ${\tilde{x}}_{i}=x_{i}-{\hat{x}}_{i}$, we have ${\tilde{x}}_{i}={\tilde{\zeta }}_{i}$. Moreover, the overall system of (10) is

(11) $$\dot{\tilde{\zeta }}=\left[I_{N}⊗\left(\overset{⌢}{A}-\overset{⌢}{L}C\right)\right]\tilde{\zeta }$$

where $\tilde{\zeta }={\left[\begin{array}{ccc} {\tilde{\zeta }}_{1}^{T} & \ldots & {\tilde{\zeta }}_{N}^{T} \end{array}\right]}^{T}$.

After we have obtained the state estimation of the *i*th follower agent, next, we plan to propose an FR via an interval observer by sensor measurement output. To begin with, based on the sensor measurement output of (2), we have

(12) $${\dot{y}}_{i}=CAx_{i}+CB\left(u_{i}+d_{i}\right)$$

We design an interval observer for (12) as follows:

(13) $$\begin{array}{c} {\dot{\bar{y}}}_{i}=CA{\hat{x}}_{i}+CBu_{i}+{\left(CB\right)}^{+}{\bar{d}}_{i}-{\left(CB\right)}^{-}{\underline{d}}_{i}+Q\left({\bar{y}}_{i}-y_{i}\right)\\ {\dot{\underline{y}}}_{i}=CA{\hat{x}}_{i}+CBu_{i}+{\left(CB\right)}^{+}{\underline{d}}_{i}-{\left(CB\right)}^{-}{\bar{d}}_{i}+Q\left({\underline{y}}_{i}-y_{i}\right) \end{array}$$

where ${\hat{x}}_{i}$ is the state estimation provided by UIO (9), and $Q\in R^{p\times p}$ is an arbitrary Metzler and Hurwitz matrix. The error equation of the interval observer is derived from (12) and (13)

(14) $$\begin{array}{c} {\dot{\bar{\varepsilon }}}_{i}=Q{\bar{\varepsilon }}_{i}-CA{\tilde{x}}_{i}+{\left(CB\right)}^{+}{\bar{d}}_{i}-{\left(CB\right)}^{-}{\underline{d}}_{i}-CBd_{i}\\ {\dot{\underline{\varepsilon }}}_{i}=Q{\underline{\varepsilon }}_{i}-CA{\tilde{x}}_{i}+CBd_{i}-{\left(CB\right)}^{+}{\underline{d}}_{i}+{\left(CB\right)}^{-}{\bar{d}}_{i} \end{array}$$

where ${\bar{\varepsilon }}_{i}={\bar{y}}_{i}-y_{i}$ and ${\underline{\varepsilon }}_{i}=y_{i}-{\underline{y}}_{i}$.

**Lemma** **2** ([39])**.** *Suppose that ${\underline{x}}_{i}\left(t\right)\leq x_{i}\left(t\right)\leq {\bar{x}}_{i}\left(t\right)$ with $x_{i}\left(t\right)\in R^{n}$, ${\bar{x}}_{i}\left(t\right)\in R^{n}$ and ${\underline{x}}_{i}\left(t\right)\in R^{n}$, and $W\in R^{m\times n}$ is a constant matrix; then $W^{+}{\underline{x}}_{i}\left(t\right)-W^{-}{\bar{x}}_{i}\left(t\right)\leq Wx_{i}\left(t\right)\leq W^{+}{\bar{x}}_{i}\left(t\right)-W^{-}{\underline{x}}_{i}\left(t\right)$.*

**Lemma** **3** ([39])**.** *For a dynamic system $\dot{x}=\Theta x+\rho \left(t\right)$, if Θ is Metzler, x(0)≥0 and ρ(t)≥0, then x(t)≥0(t≥0).*

**Lemma** **4.** *Under Assumptions 1 and 5, system (13) is an interval observer of system (12) such that ${\underline{y}}_{i}\left(t\right)\leq y_{i}\left(t\right)\leq {\bar{y}}_{i}\left(t\right)$ holds for all t≥0, if the initial values are set as ${\bar{y}}_{i}\left(0\right)=C^{+}{\bar{x}}_{i}-C^{-}{\underline{x}}_{i}$ and ${\underline{y}}_{i}\left(0\right)=C^{+}{\underline{x}}_{i}-C^{-}{\bar{x}}_{i}$, and Q is a Hurwitz and Metzler matrix chosen arbitrarily.*

**Proof:** Refer to Appendix B.

Since ${\underline{y}}_{i}\left(t\right)\leq y_{i}\left(t\right)\leq {\bar{y}}_{i}\left(t\right)$, there exists a time-varying vector $\alpha_{i}\left(t\right)={\left[\alpha_{i1}\left(t\right),\ldots ,\alpha_{ip}\left(t\right)\right]}^{T}$$\in R^{p}$, satisfying $0\leq \alpha_{ij}\left(t\right)\leq 1$, such that

(15) $$y_{i}=\mathrm{diag}\left({\overset{⌣}{y}}_{i}\right)\alpha_{i}+{\underline{y}}_{i}$$

where ${\overset{⌣}{y}}_{i}={\bar{y}}_{i}-{\underline{y}}_{i}$. In addition, (15) gives

(16) $$\alpha_{i}=\left[\begin{array}{c} {\mathrm{diag}}^{-1}\left({\overset{⌣}{y}}_{i}+\gamma_{i}\right)-\mathrm{diag}\left(\gamma_{i}\right) \end{array}\right]\left(y_{i}-{\underline{y}}_{i}\right)$$

where $\gamma_{i}\in R^{p}$ and $\gamma_{i,j}=1$ if ${\overset{⌣}{y}}_{i,j}=0$; otherwise, $\gamma_{i,j}=0\left(j=1,\ldots ,p\right)$. Here $\gamma_{i,j}$ and ${\overset{⌣}{y}}_{i,j}$ are the *j*th element of the vectors of $\gamma_{i}$ and ${\overset{⌣}{y}}_{i}$, respectively. Differentiating (15), we obtain

(17) $${\dot{y}}_{i}=\mathrm{diag}\left(\dot{\overset{⌣}{y}}\right)\alpha_{i}+\mathrm{diag}\left({\overset{⌣}{y}}_{i}\right){\dot{\alpha }}_{i}+\dot{{\underline{y}}_{i}}$$

It follows from (13) that

(18) $${\dot{\overset{⌣}{y}}}_{i}=\phi_{1}=Q{\overset{⌣}{y}}_{i}+\left|CB\right|{\overset{⌣}{d}}_{i}$$

where ${\overset{⌣}{d}}_{i}={\bar{d}}_{i}-{\underline{d}}_{i}$. In addition, the second equation of (13) is

(19) $${\dot{\underline{y}}}_{i}=CA{\hat{x}}_{i}+CBu_{i}+\phi_{2}$$

where $\phi_{2}={\left(CB\right)}^{+}{\underline{d}}_{i}-{\left(CB\right)}^{-}{\bar{d}}_{i}+Q\left({\underline{y}}_{i}-y_{i}\right)$. Now, substituting (18) and (19) into (17) yields

(20) $${\dot{y}}_{i}=\mathrm{diag}\left(\phi_{1}\right)\alpha_{i}+\mathrm{diag}\left({\overset{⌣}{y}}_{i}\right){\dot{\alpha }}_{i}+CA{\hat{x}}_{i}+CBu_{i}+\phi_{2}$$

Comparing (12) with (20), we obtain

(21) $$CBd_{i}=\mathrm{diag}\left(\phi_{1}\right)\alpha_{i}+\mathrm{diag}\left({\overset{⌣}{y}}_{i}\right){\dot{\alpha }}_{i}-CA{\tilde{x}}_{i}+\phi_{2}$$

Using the rank condition in *Assumption 1* again, (21) gives

(22) $$d_{i}={\left(CB\right)}^{†}\left[\mathrm{diag}\left(\phi_{1}\right)\alpha_{i}+\mathrm{diag}\left({\overset{⌣}{y}}_{i}\right){\dot{\alpha }}_{i}+\phi_{2}-CD{\tilde{x}}_{i}\right]$$

By setting ${\tilde{x}}_{i}=0$ in (22), an FR is provided as

(23) $${\hat{d}}_{i}={\left(CB\right)}^{†}\left[\mathrm{diag}\left(\phi \right)\alpha_{i}+\mathrm{diag}\left({\overset{⌣}{y}}_{i}\right){\hat{\dot{\alpha }}}_{i}+\phi_{2}\right]$$

where ${\hat{\dot{\alpha }}}_{i}$ represents the exact finite-time estimate of ${\dot{\alpha }}_{i}$ provided by the differentiator [40]:

(24) $$\left\{\begin{array}{cc} {\dot{\xi }}_{1,ij} & =\kappa_{1,ij}=-\rho_{1,ij}{\left|\xi_{1,ij}-\alpha_{ij}\right|}^{1/2}\mathrm{sign}\left(\xi_{1,ij}-\alpha_{ij}\right)+\xi_{2,ij}\\ {\dot{\xi }}_{2,ij} & =-\rho_{2,ij}\;\mathrm{sign}\left(\xi_{2,ij}-\kappa_{1,ij}\right),\;\left(j=1,\ldots ,p\right) \end{array}\right.$$

where $\rho_{1,ij}>0$ and $\rho_{2,ij}>0$ are positive scalar gains. $\xi_{2,ij}$ is the identical estimation of ${\dot{\alpha }}_{ij}$ in a finite time. Therefore, ${\hat{\dot{\alpha }}}_{i}={\left[\xi_{2,i1}\;\ldots \;\xi_{2,ip}\right]}^{T}$ is the identical estimation of ${\dot{\alpha }}_{i}$ in a finite time. That is, ${\dot{\alpha }}_{i}\left(t\right)\equiv {\hat{\dot{\alpha }}}_{i}\left(t\right)$; then it follows from (22) and (23) that

(25) $${\tilde{d}}_{i}=-{\left(CB\right)}^{†}CA{\tilde{x}}_{i}$$

The FR offered by (23) is able to estimate the actual unknown input $d_{i}$ asymptotically. In fact, $\lim_{t\to \infty }{\tilde{d}}_{i}\left(t\right)=-{\left(CB\right)}^{†}CA\lim_{t\to \infty }{\tilde{x}}_{i}\left(t\right)=0$, where ${\tilde{d}}_{i}=d_{i}-{\hat{d}}_{i}$.

**Remark** **3.** *The proposed FR (23) shows several advantages. First, it provides an asymptotic convergence estimation of the actual fault. Secondly, it does not rely on derivative information of the fault. Thirdly, the FR decouples the control input $u_{i}$ of the ith follower; thereby, an FR-based compensating controller is effectively derived.*

## 4. DO Design and DO-Based Fault-Tolerant Control Protocol

### 4.1. DO Design

In this section, for each follower agent, we try to design a DO such that each follower agent can obtain the state estimation of the leader’s. And then, a DO-based control protocol scheme is developed.

For the *i*th follower agent, consider

(26) $$\begin{array}{c} {\dot{\overset{⌢}{x}}}_{0,i}=A{\overset{⌢}{x}}_{0,i}+M\left[\sum_{j=1}^{N}a_{ij}\left({\overset{⌢}{x}}_{0,i}-{\overset{⌢}{x}}_{0,j}\right)+a_{i0}\left({\overset{⌢}{x}}_{0,i}-{\hat{x}}_{0}\right)\right] \end{array}$$

where ${\hat{x}}_{0}$ is provided by a local observer (4). Denote ${\overset{⌣}{x}}_{0,i}=x_{0}-{\overset{⌢}{x}}_{0,i}$; then the dynamic system of ${\overset{⌣}{x}}_{0,i}$ is obtained by subtracting (26) from (3)

(27) $$\begin{array}{c} {\dot{\overset{⌣}{x}}}_{0,i}=A{\overset{⌣}{x}}_{0,i}-Ma_{i0}{\tilde{x}}_{0}+Bu_{0}+M\left[\sum_{j=1}^{N}a_{ij}\left({\overset{⌣}{x}}_{0,i}-{\overset{⌣}{x}}_{0,j}\right)+a_{i0}{\overset{⌣}{x}}_{0,i}\right] \end{array}$$

The overall system of (27) is

(28) $$\begin{array}{c} {\dot{\overset{⌣}{x}}}_{0}=\left(I_{N}⊗A+M⊗H\right){\overset{⌣}{x}}_{0}+\left(1_{N}⊗B\right)u_{0}-\left(D1_{N}⊗M\right){\tilde{x}}_{0} \end{array}$$

where ${\overset{⌣}{x}}_{0}={\left[{\overset{⌣}{x}}_{0,1}^{T}\;\ldots \;{\overset{⌣}{x}}_{0,N}^{T}\right]}^{T}$.

**Remark** **4.** *Note that the leader’s control input $u_{0}$ is local information to the leader and only those followers which are connected to the leader directly can access the information of the leader’s. Therefore, in view of the distributed structure, the DO cannot involve the $u_{0}$ in Equation (26).*

**Remark** **5.** *Generally, since not every follower can directly obtain the state information of the leader’s referring to the communication topology, constructing distributed control protocols becomes the major method for MAS consensus problems [41,42,43,44]. In the present paper, a DO-based control protocol is developed, and the distributed feature is majorly reflected by the DO. Therefore, the DO (26) plays an important role in the designs.*

**Remark** **6.** *It is worth noting the fundamental distinction between fully distributed and partially centralized (DO-based) control architectures. Fully distributed protocols directly embed the communication topology within the feedback control loop. Under severe local disturbances, this continuous state exchange can exacerbate fault propagation and communication burdens. In contrast, the proposed partially centralized scheme decouples the network topology from the local controller by strictly confining distributed interactions to the DO (26). Consequently, local state estimation and fault-tolerant control are executed independently, making it highly suitable for practical leader–following MASs (2) and (3).*

### 4.2. DO-Based Fault-Tolerant Control Protocol

In this subsection, we design a DO-based fault-tolerant control protocol to ensure that the multi-agent system reaches consensus according to Definition 1.

Define $\chi_{i}=x_{i}-x_{0}$; then its dynamic is

(29) $$\dot{\chi_{i}}=A\chi_{i}+B\left(u_{i}+d_{i}-u_{0}\right)$$

Now, based on the DO (26), the UIO (9) and the FR (23), we propose an observer-based state feedback control scheme with fault compensation by

(30) $$u_{i}=K{\hat{\chi }}_{i}-{\hat{d}}_{i}$$

where ${\hat{\chi }}_{i}={\hat{x}}_{i}-{\overset{⌢}{x}}_{0,i}$; then the closed-loop system of (29) is

(31) $$\begin{array}{c} {\dot{\chi }}_{i}=\left(A+BK\right)\chi_{i}-BK{\tilde{x}}_{i}+BK{\overset{⌣}{x}}_{0,i}+B{\tilde{d}}_{i}-Bu_{0} \end{array}$$

Substitute (25) into (31), and noticing that ${\tilde{x}}_{i}={\tilde{\zeta }}_{i}$, we have

(32) $$\begin{array}{c} {\dot{\chi }}_{i}=\left(A+BK\right)\chi_{i}+BK{\overset{⌣}{x}}_{0,i}-B\left({\left(CB\right)}^{†}CA+K\right){\tilde{\zeta }}_{i}-Bu_{0} \end{array}$$

The overall system of (32) is

(33) $$\begin{array}{c} \begin{array}{cc} \dot{\chi }= & \left[\begin{array}{c} I_{N}⊗\left(A+BK\right) \end{array}\right]\chi +\left(I_{N}⊗BK\right){\overset{⌣}{x}}_{0}-\\ & \left[\begin{array}{c} I_{N}⊗B\left({\left(CB\right)}^{†}CA+K\right) \end{array}\right]\tilde{\zeta }-\left(I_{N}⊗B\right)\left(1_{N}⊗I_{n}\right)u_{0} \end{array} \end{array}$$

**Figure 1:**
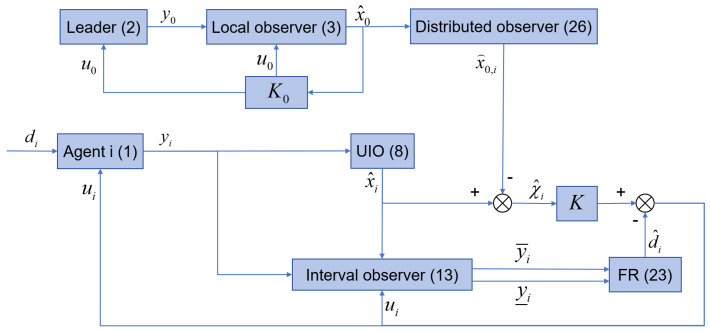
DO-based fault-tolerant control protocol mechanism.

Now, the DO-based control protocol mechanism can be illustrated by Figure 1.

**Remark** **7.** *Through a series of procedures, a DO-based control protocol mechanism for MASs (2) and (3), which is illustrated clearly by Figure 1, is developed. The DO-based control protocol mechanism is accomplished by several constructions. Firstly, a DO (26) is developed such that each follower can obtain the asymptotic convergence state estimation of the leader’s. Secondly, a local UIO (9) is to ensure that each follower agent reaches an asymptotic convergence in state estimation by eliminating the negative effect of the fault. Thirdly, through a sensor interval observer, an FR method (23) is given. The FR proposed is capable of estimating the actual fault. In addition, it decouples the control input $u_{i}$. Finally, for each follower agent, a controller (30) is constructed by using the estimated information provided by the DO (26), the local UIO (9) and the FR (23). And because of the DO, the form of the controller (30) looks like a local one rather than a distributed one. In fact, the proposed DO-based control protocol consists of two parts. One is the combination of observer constructions (UIO (9) together with FR (23), and DO (26)). The other is the feedback controller (30). It should be emphasized that the proposed control protocol is a distributed one and the distributed feature has been moved on DO, resulting in the controller being in centralized form. Next, we will prove that the DO-based consensus mechanism is able to fulfill the task of asymptotic convergence MAS consensus.*

Denote $\psi ={\left[\begin{array}{cccc} \chi^{T} & {\overset{⌣}{x}}_{0}^{T} & {\tilde{\zeta }}^{T} & {\tilde{x}}_{0}^{T} \end{array}\right]}^{T}$; then by (3), (5), (11), (28) and (33), we obtain

(34) $$\dot{\psi }=\Xi \psi +\bar{B}u_{0}$$

where

$$\Xi =\left[\begin{array}{cccc} I_{N}⊗\left(A+BK\right) & I_{N}⊗BK & -I_{N}⊗B\left({\left(CB\right)}^{†}CA+K\right) & 0\\ 0 & I_{N}⊗A+M⊗H & 0 & -D1_{N}⊗M\\ 0 & 0 & I_{N}⊗\left(\overset{⌢}{A}-\overset{⌢}{L}C\right) & 0\\ 0 & 0 & 0 & A-LC \end{array}\right]$$

$$\bar{B}=\left[\begin{array}{c} -1⊗B\\ 1⊗B\\ 0\\ 0 \end{array}\right]$$

Before we present the main result, we further define

(35) $$\Pi =\left[\begin{array}{ccccc} \Pi_{11} & \Pi_{12} & \Pi_{13} & 0 & \Pi_{15}\\ {}* & \Pi_{22} & 0 & \Pi_{24} & \Pi_{25}\\ {}* & * & \Pi_{33} & 0 & 0\\ {}* & * & * & \Pi_{44} & 0\\ {}* & * & * & * & \Pi_{55} \end{array}\right]$$

where

$$\begin{array}{cc} \Pi_{11} & =I_{N}⊗\left(P_{1}A+A^{T}P_{1}+2P_{1}BK+I_{n}\right)\\ \Pi_{12} & =I_{N}⊗P_{1}BK\\ \Pi_{13} & =-I_{N}⊗\left(P_{1}B{\left(CB\right)}^{†}CA+P_{1}BK\right)\\ \Pi_{15} & =-1_{N}⊗P_{1}BK_{0}\\ \Pi_{22} & =I_{N}⊗\left(P_{2}A+A^{T}P_{2}+I_{n}\right)+H⊗Y+H^{T}⊗Y^{T}\\ \Pi_{24} & =-D1_{N}⊗Y\\ \Pi_{25} & =1_{N}⊗P_{2}B\\ \Pi_{33} & =I_{N}⊗\left(P_{3}\overset{⌢}{A}+{\overset{⌢}{A}}^{T}P_{3}-\overset{⌢}{U}C-C^{T}{\overset{⌢}{U}}^{T}\right)\\ \Pi_{44} & =P_{4}A+A^{T}P_{4}-UC-C^{T}U^{T}+I_{n}\\ \Pi_{55} & =-\gamma^{2}I_{p} \end{array}$$

**Theorem** **1.** *Under Assumptions 1–4 and given that Π≺0 has solutions for $P_{1}≻0$, $P_{2}≻0$, $P_{3}≻0$, $P_{4}≻0$, Y, U, $\overset{⌢}{U}$ and min γ, the leader–following consensus control of MAS (2) and (3) can be fulfilled under the DO-based control protocol (30) in a sense of H-infinity stability, if we set $M=P_{2}^{-1}Y$, $\overset{⌢}{L}=P_{3}^{-1}\overset{⌢}{U}$ and $L=P_{4}^{-1}U$.*

**Proof** **of Theorem 1.** Consider Lyapunov function $V=\psi^{T}P\psi$, where $P=\mathrm{diag}(P_{1},P_{2},P_{3},P_{4})$. Then, the derivative of *V* along the trajectories of (34) is

(36) $$\dot{V}+\psi^{T}\psi -\gamma^{2}u_{0}^{T}u_{0}=\left[\begin{array}{cc} \psi^{T} & u_{0}^{T} \end{array}\right]\Pi \left[\begin{array}{c} \psi \\ u_{0} \end{array}\right]$$

where Π is defined by (35). Now, by (36), we have $∥\psi ∥\leq \gamma ∥u_{0}∥$ with the minimal γ, which means that dynamic system (34) is H-infinity stable satisfying the performance index of norm, and this completes the proof of Theorem 1.    □

In order to calculate the parameters which are required in the designs, we formulate the following Riccati equations:

(37) $$I_{N}⊗\left(A^{T}P_{1}+P_{1}A-2P_{1}BB^{T}P_{1}\right)=-I_{N}⊗Q_{1}$$

Subsequently, to determine the state feedback gain matrix *K* in (29), $K_{0}$ in (7), *L* in (4), $\overset{⌢}{L}$ in (9), and *M* in (26), we offer the following Algorithm 1.
**Algorithm 1** Calculation Process of Gain Matrix**Input:** System matrices *A*, *B*, *C*; degree matrices D; Generalized Laplacian matrix H.
**Output:** DO gain matrix *M*; observer gain matrices *L*, $\overset{⌢}{L}$; and state feedback gain matrices *K*, $K_{0}$.
 1:Solve algebraic Riccati equations (AREs) (37) to obtain $P_{1}$; then compute $K=-B^{T}P_{1}$. 2:Solve LMI Π≺0 to obtain $P_{2}$, $P_{3}$, $P_{4}$, *Y*, *U*, $\overset{⌢}{U}$ and γ; then, set $K_{0}=-B^{T}P_{2}$, $M=YP_{2}^{-1}$, $L=UP_{4}^{-1}$ and $\overset{⌢}{L}=\overset{⌢}{U}P_{3}^{-1}$. 3:**Return***M*, *L*, $\overset{⌢}{L}$, *K*, $K_{0}$.


## 5. Simulation Results

In this section, two real systems are taken as simulation examples to verify the effectiveness of the DO-based control protocol.

**Figure 2:**
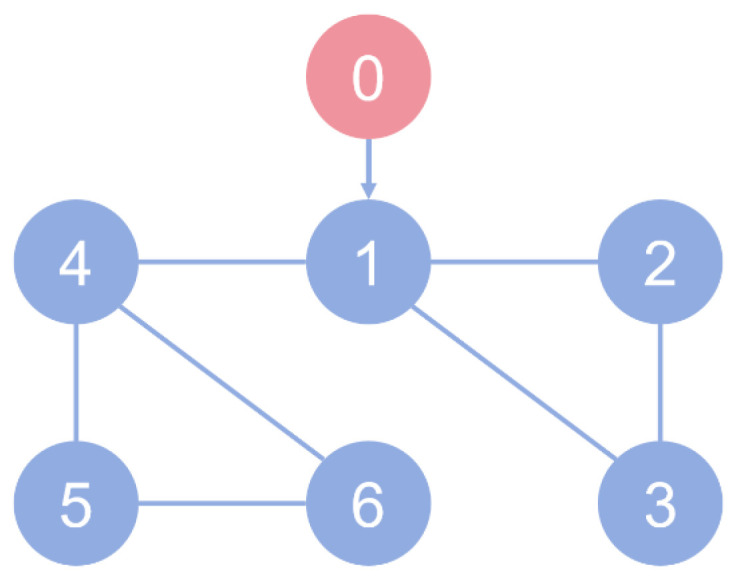
Communication topology.

**Example** **1.** *Consider a multi-agent system comprising a single leader and six followers, where each node is modeled as a dual-mass-spring-damper mechanism. The Communication topology is displayed in Figure 2, where the leader state can be only accessed by follower agent 1, and the communication graph for followers is a undirected one. Based on Figure 2, the Laplacian matrix is*

$$L=\left[\begin{array}{cccccc} 4 & -1 & -1 & -1 & 0 & 0\\ -1 & 2 & -1 & 0 & 0 & 0\\ -1 & -1 & 2 & 0 & 0 & 0\\ -1 & 0 & 0 & 3 & -1 & -1\\ 0 & 0 & 0 & -1 & 2 & -1\\ 0 & 0 & 0 & -1 & -1 & 2 \end{array}\right]$$

**Figure 3:**
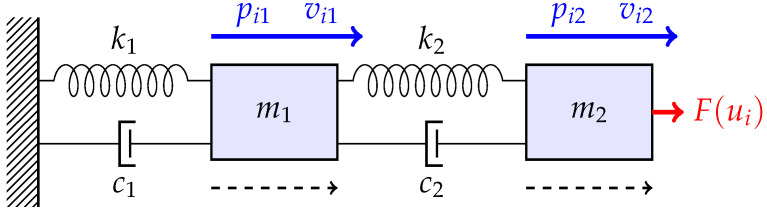
Two-degree-of-freedom (2-DOF) mass-spring-damper system.

*Each agent comprises two mass blocks and two springs as shown in Figure 3. The system state is defined as $x_{i}={\left[\begin{array}{cccc} p_{i1} & v_{i1} & p_{i2} & v_{i2} \end{array}\right]}^{T}$, where $p_{i1}$ and $v_{i1}$ denote the displacement and velocity of mass 1, and $p_{i2}$ and $v_{i2}$ indicate the displacement and velocity of mass 2. This system is controlled by force F acting on $m_{2}$, which is the state input value $u_{i}$ in (38). Then, the dynamic takes the following form:*

(38) $$\begin{array}{cc} {\dot{x}}_{i} & =\left[\begin{array}{cccc} 0 & 1 & 0 & 0\\ -\frac{k_{1}+k_{2}}{m_{1}} & -\frac{c_{1}+c_{2}}{m_{1}} & \frac{k_{2}}{m_{1}} & \frac{c_{2}}{m_{1}}\\ 0 & 0 & 0 & 1\\ \frac{k_{2}}{m_{2}} & \frac{c_{2}}{m_{2}} & -\frac{k_{2}}{m_{2}} & -\frac{c_{2}}{m_{2}} \end{array}\right]x_{i}+\left[\begin{array}{c} 0\\ 0\\ 0\\ \frac{1}{m_{2}} \end{array}\right]\left(u_{i}+\omega_{i}\right)\\ y_{i} & =\left[\begin{array}{cccc} 1 & 1 & 0 & 0\\ 0 & 0 & 1 & 0\\ 0 & 0 & 0 & 1 \end{array}\right]x_{i} \end{array}$$

*where* $m_{1}=10.0$ kg*,* $m_{2}=5.0$ kg; $k_{1}=20.0$ N/m *and* $k_{2}=15.0$ N/m *indicate the spring stiffness of two springs*; $c_{1}=12.0\;$ N·s/m *and* $c_{2}=8.0$ N·s/m *are the damping coefficients. It is clear that the system (38) comes from the form of (2). To construct MUI, we assume that* *$\eta_{i}=\left\{\begin{array}{cc} 0.5\cdot 1_{1\times 6}, & t\leq 15\\ 1_{1\times 6}, & t>15 \end{array}\right.\;\;,\;f_{a,i}=\left\{\begin{array}{cc} 2\cdot 1_{1\times 6}, & t\leq 15\\ 2\sin\left(3t\right)\cdot 1_{1\times 6}, & t>15 \end{array}\right.$, and $\omega_{i}\left(t\right)\in R^{2}$ indicate the fault in system (2):*

$$\begin{array}{cc} \omega_{1}\left(t\right) & =1.2\sin(0.8t)+0.6\sin(4t)\\ \omega_{2}\left(t\right) & =(1.0+0.5\sin(0.2t))\sin(2t)\\ \omega_{3}\left(t\right) & =1.8\sin(t+0.8\sin(0.5t))\\ \omega_{4}\left(t\right) & =\left\{\begin{array}{cc} 1.5\sin(t), & t\leq 15\\ 1.5\sin(t)+0.4\sin(5(t-15)), & t>15 \end{array}\right.\\ \omega_{5}\left(t\right) & =(1.0+0.8\tanh(3\sin(0.2t)))\sin(2t)\\ \omega_{6}\left(t\right) & =0.6\sin\left(t\right)+0.6\sin\left(\sqrt{2}t\right)+0.6\sin\left(\sqrt{5}t\right) \end{array}$$

*By solving the ARE (37) with $Q_{1}=2*I_{N}$, the positive definite matrix and the correlated state feedback gain matrices are obtained as follows:*

$$\begin{array}{cccc} P_{1} & =\left[\begin{array}{cccc} 33.7621 & 7.5022 & 3.3705 & 5.6305\\ 7.5022 & 15.3939 & 1.5684 & 9.4105\\ 3.3705 & 1.5684 & 28.1465 & 4.0103\\ 5.6305 & 9.4105 & 4.0103 & 11.7391 \end{array}\right] & \;K & =\left[\begin{array}{cccc} 1.1261 & 1.8821 & 0.8021 & 2.3478 \end{array}\right] \end{array}$$

*Next, we solve LMI Π≺0 to obtain $P_{2},P_{3},P_{4},U,\overset{⌢}{U}$ and Y as:*

$$\begin{array}{cccc} P_{2} & =\left[\begin{array}{cccc} 1541.6 & 353.8 & -841.7 & -14.8\\ 353.8 & 206.6 & -210.4 & -52.3\\ -841.7 & -210.4 & 484.9 & 17.2\\ -14.8 & -52.3 & 17.2 & 40.8 \end{array}\right] & \;K_{0} & =\left[\begin{array}{cccc} 2.964 & 10.461 & -3.447 & -8.163 \end{array}\right] \end{array}$$

$$\begin{array}{cccc} P_{3} & =\left[\begin{array}{cccc} 2903.4 & 325.7 & 50.8 & -1553.4\\ 325.7 & 2218.0 & -955.6 & 194.4\\ 50.8 & -955.6 & 1944.8 & 1910.7\\ -1553.4 & 194.4 & 1910.7 & 4041.4 \end{array}\right] & \;\overset{⌢}{U} & =\left[\begin{array}{ccc} 6024 & -9206 & 9691\\ 1257 & -2357 & -7320\\ -4792 & 14,033 & 4883\\ -1087 & 4883 & -2555 \end{array}\right]\\ P_{4} & =\left[\begin{array}{cccc} 3733.2 & 93.2 & -23.2 & -698.9\\ 93.2 & 1850.4 & 3.5 & 351.8\\ -23.2 & 3.5 & 2547.3 & -3.1\\ -698.9 & 351.8 & -3.1 & 2734.4 \end{array}\right] & \;U & =\left[\begin{array}{ccc} -831.6 & 1061.9 & 4243.3\\ -1555.1 & 511.1 & -91.6\\ 1273.6 & 1725.7 & -2496.7\\ 4156.7 & -2496.7 & -2306.6 \end{array}\right] \end{array}$$

$$\begin{array}{cc} Y & =\left[\begin{array}{cccc} 0.1000 & 0.1000 & 0.1000 & 0.4371\\ 0.1000 & 0.1000 & 0.1000 & 0.1000\\ 0.1000 & 0.1000 & 0.1000 & 0.1000\\ 0.6829 & 0.1000 & 0.1000 & 0.1000 \end{array}\right] \end{array}$$

*Moreover, the minimum parameter value γ=2.6187 is obtained through Algorithm 1; we have*

$$\begin{array}{cccc} L & =\left[\begin{array}{ccc} 0.1287 & 0.1006 & 1.0207\\ -1.1719 & 0.4494 & 0.0123\\ 0.5049 & 0.6766 & -0.9716\\ 1.7044 & -0.9444 & -0.5853 \end{array}\right] & \;\overset{⌢}{L} & =\left[\begin{array}{ccc} 24.4191 & -52.5562 & 10.6957\\ -27.7509 & 62.1136 & -11.9467\\ -50.4286 & 113.3521 & -14.2307\\ 34.2928 & -75.5695 & 10.7813 \end{array}\right]\\ M & =\left[\begin{array}{cccc} -0.0007 & 0.0029 & 0.0029 & 0.0075\\ 0.0139 & 0.0036 & 0.0036 & 0.0032\\ 0.0038 & 0.0066 & 0.0066 & 0.0144\\ 0.0326 & 0.0053 & 0.0053 & 0.0032 \end{array}\right] \end{array}$$

*For the 2-DOF mass-spring-damper system, the leader’s initial state is set as $x_{0}\left(0\right)={\left[2\;0\;2\;0\right]}^{T}$, and the followers’ initial states are $x_{1}\left(0\right)={\left[-2\;0.4\;1.6\;0.0\right]}^{T}$, $x_{2}\left(0\right)={\left[-2.4\;0.0\;-2\;0.0\right]}^{T}$, $x_{3}\left(0\right)={\left[2\;0.0\;2.4\;0.0\right]}^{T}$, $x_{4}\left(0\right)={\left[2.4\;0.0\;4\;0.0\right]}^{T}$, $x_{5}\left(0\right)={\left[-2.8\;0.0\;2.4\;0.0\right]}^{T}$ and $x_{6}\left(0\right)={\left[-2\;0.0\;1.6\;0.0\right]}^{T}$.*

**Figure 4:**
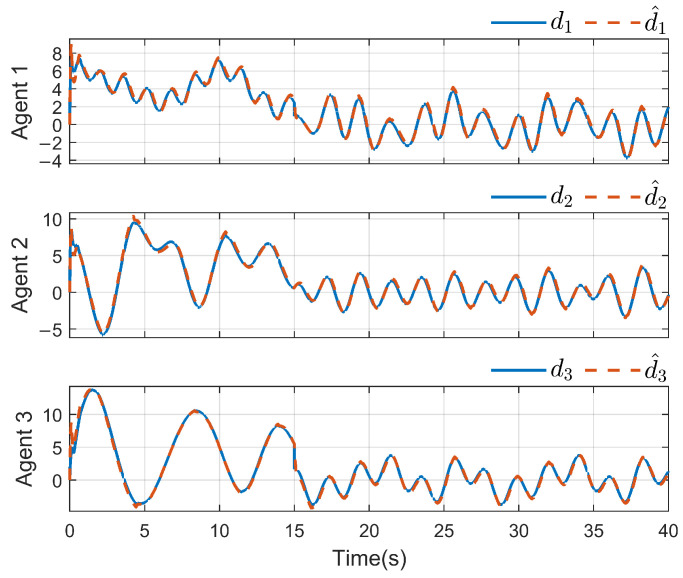
$d_{i}$ and its reconstruction ${\hat{d}}_{i}$(i=1,2,3).

**Figure 5:**
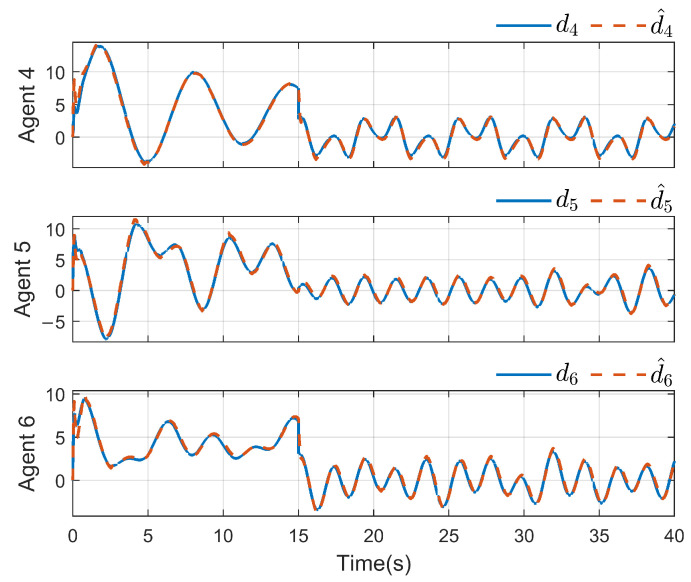
$d_{i}$ and its reconstruction ${\hat{d}}_{i}$(i=4,5,6).

**Figure 6:**
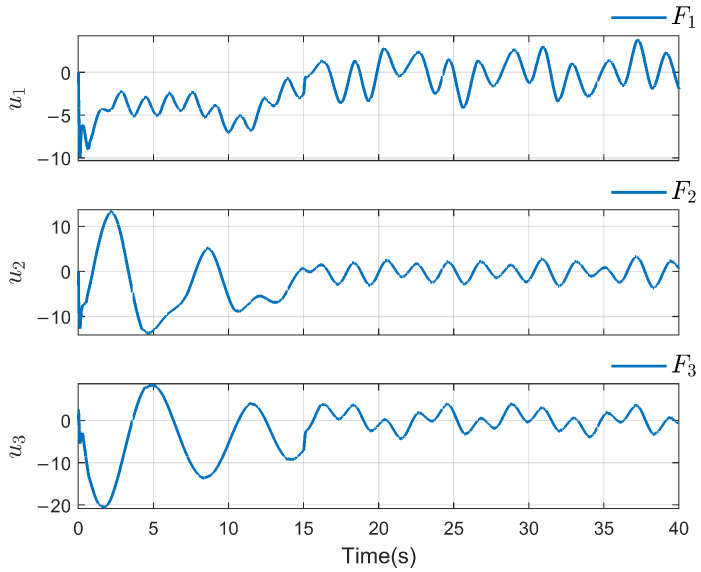
Control input $u_{i}$(i=1,2,3).

**Figure 7:**
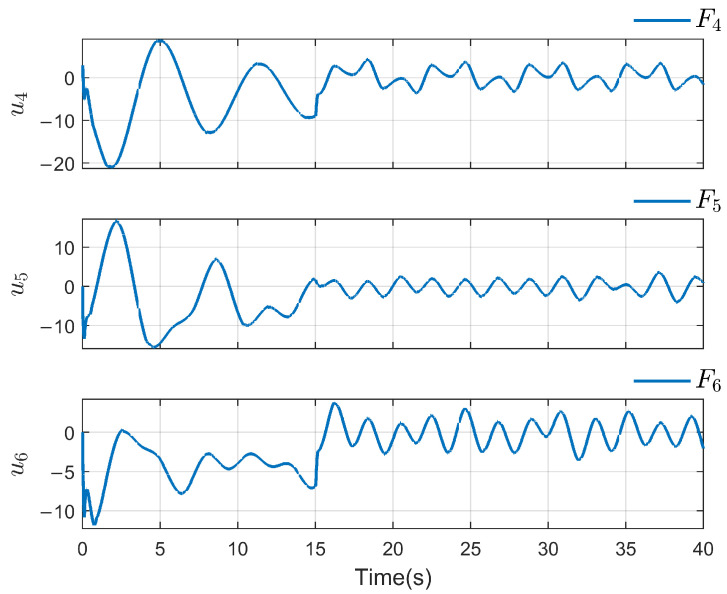
Control input $u_{i}$(i=4,5,6).

**Figure 8:**
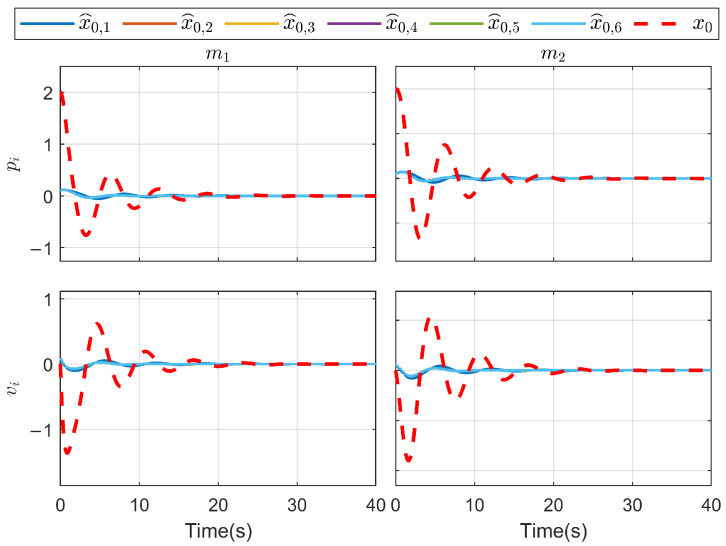
DO estimation for each agent.

**Figure 9:**
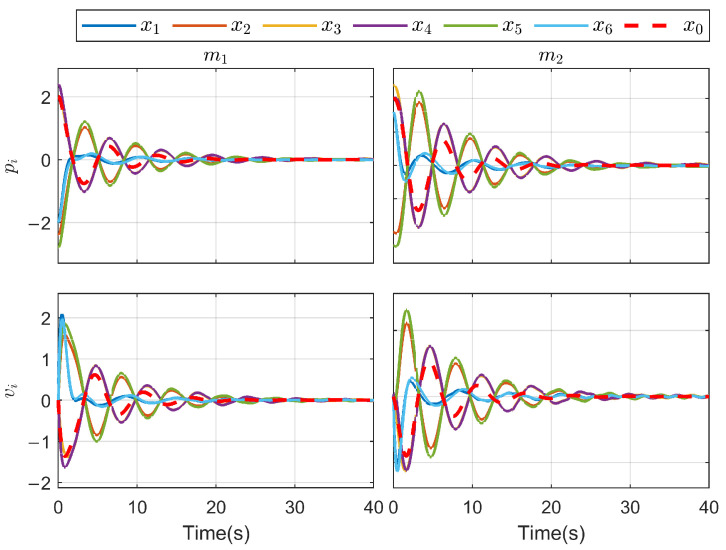
State trajectory for each agent.

*To verify the effectiveness of the proposed method, the detailed simulation results are presented as follows. Figure 4 and Figure 5 show the performance of FR, $d_{i}$ and its reconstruction ${\hat{d}}_{i}$, in which we set ${\underline{u}}_{i}={\left[-2\;-2\;-2\;-2\;-2\;-2\right]}^{T}$, ${\bar{u}}_{i}={\left[2\;2\;2\;2\;2\;2\right]}^{T}$, ${\underline{f}}_{a,i}={\left[-2\;-2\;-2\;-2\;-2\;-2\right]}^{T}$, ${\bar{f}}_{a,i}={\left[2\;2\;2\;2\;2\;2\right]}^{T}$, ${\underline{\eta }}_{i}={\left[0\;0\;0\;0\;0\;0\right]}^{T}$, ${\bar{\eta }}_{i}={\left[1\;1\;1\;1\;1\;1\right]}^{T}$, ${\underline{\omega }}_{i}={\left[-2\;-2\;-2\;-2\;-2\;-2\right]}^{T}$ and ${\bar{\omega }}_{i}={\left[2\;2\;2\;2\;2\;2\right]}^{T}$. And then, we obtain ${\underline{d}}_{i}={\left[-6\;-6\;-6\;-6\;-6\;-6\right]}^{T}$ and ${\bar{d}}_{i}={\left[8\;8\;8\;8\;8\right]}^{T}$. For the FR, $\rho_{1}=10.5$ and $\rho_{2}=8.7$ in (24), $Q=\left[\begin{array}{ccc} -10.172 & 0 & 0\\ 0 & -10.5915 & 0\\ 0 & 0 & -10 \end{array}\right]$ in (13). For the DO-based control protocol, the force F, which serves as the control input $u_{i}$, is displayed in Figure 6 and Figure 7. Now, we have the state estimation and FR of $d_{i}$ for the ith follower, and the leader’s state estimation produced in the ith follower by DO (26) in Figure 8. And under the DO-based fault-tolerant control protocol, the MAS consensus is fulfilled asymptotically for the 2-DOF mass-spring-damper system, where the displacement and velocity for masses $m_{1}$ and $m_{2}$ can be clearly seen from Figure 9.*

**Figure 10:**
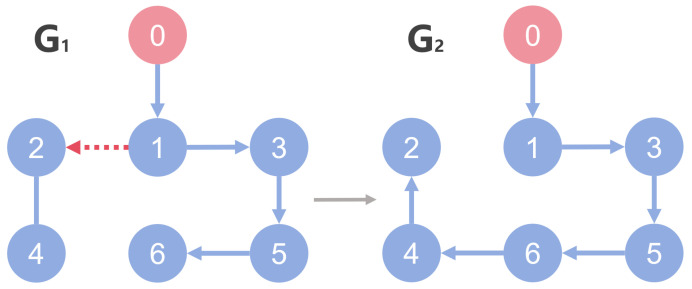
Time-varying switching topology.

**Example** **2.** *Consider a time-varying switching topology scenario described in Figure 10, and the form as follows:*

$$A=\left[\begin{array}{cc} 0 & 1.0\\ -2.5 & -1.0 \end{array}\right],\;B=\left[\begin{array}{c} 0\\ 0.25 \end{array}\right],\;C=\left[\begin{array}{cc} 1 & 0\\ 0 & 1 \end{array}\right]$$

*Assuming that Agent 2 receives information from Agent 1 within the periodic time, when Agent 2 cannot obtain the state of Agent 1, the topological frame is $G_{1}$ as shown in Figure 10; otherwise, the topological frame is $G_{2}$. To simulate the dynamic disconnection and reconnection of these communication links, a piecewise constant switching signal $\sigma \left(t\right):\left[0,30\right)\to \left\{G_{1},G_{2}\right\}$ is introduced. The network topology switches periodically every 5 s, which is formulated as:*

$$\sigma \left(t\right)=\left\{\begin{array}{cc} G_{1}, & t\in [10k,10k+5)\\ G_{2}, & t\in [10k+5,10(k+1)) \end{array}\right.,\;k\in N$$

*Under this switching mechanism, the DO design for the ith follower (26) is naturally extended by incorporating the time-varying adjacency weight $a_{ij}^{\sigma (t)}$:*

$$\begin{array}{c} {\dot{\overset{⌢}{x}}}_{0,i}=A{\overset{⌢}{x}}_{0,i}+M\left[\sum_{j=1}^{N}a_{ij}^{\sigma (t)}\left({\overset{⌢}{x}}_{0,i}-{\overset{⌢}{x}}_{0,j}\right)+a_{i0}\left({\overset{⌢}{x}}_{0,i}-{\hat{x}}_{0}\right)\right] \end{array}$$

*For different physical models, we define the same $\eta_{i}$ and $f_{a,i}$ by a DO-based fault-tolerant control protocol. To solve ARE (37) and LMI Π≺0, the positive definite matrix and the correlated state feedback gain matrices are obtained as follows:*

$$\begin{array}{cccccc} P_{1} & =\left[\begin{array}{cc} 29.1241 & 1.9089\\ 1.9089 & 4.6203 \end{array}\right] & \;P_{2} & =\left[\begin{array}{cc} 11.0282 & 0.1897\\ 0.1897 & 2.5146 \end{array}\right] & \;P_{3} & =\left[\begin{array}{cc} 995.9838 & 958.5875\\ 958.5875 & 999.9381 \end{array}\right]\\ K & =\left[\begin{array}{cc} 0.9545 & 2.3101 \end{array}\right] & \;K_{0} & =\left[\begin{array}{cc} -0.0474 & -0.6286 \end{array}\right] & \;\overset{⌢}{U} & =\left[\begin{array}{cc} 1872.0 & 9355.0\\ 9355.0 & 4823.0 \end{array}\right]\\ P_{4} & =\left[\begin{array}{cc} 999.9493 & 999.9096\\ 999.9096 & 999.9828 \end{array}\right] & \;Y & =\left[\begin{array}{cc} 0.1 & 0.1856\\ 0.1 & 0.1 \end{array}\right] & \;L & =\left[\begin{array}{cc} -17,748 & -17,770\\ 17,744 & 17,768 \end{array}\right]\\ U & =\left[\begin{array}{cc} -4972.4 & -2968.6\\ -2968.6 & -962.3 \end{array}\right] & \;M & =\left[\begin{array}{cc} 0.0084 & 0.0162\\ 0.0391 & 0.0385 \end{array}\right] & \;\overset{⌢}{L} & =\left[\begin{array}{cc} 126.5875 & 61.4250\\ -111.9966 & -54.0617 \end{array}\right] \end{array}$$

*The minimum parameter value γ=2.0578 as obtained by Algorithm 1, the leader’s initial state is set for $x_{0}\left(0\right)={\left[2\;0\right]}^{T}$, and the followers’ initial states are $x_{1}\left(0\right)={\left[-2\;0.4\right]}^{T}$, $x_{2}\left(0\right)={\left[-2.4\;0.0\right]}^{T}$, $x_{3}\left(0\right)={\left[2\;0.0\right]}^{T}$, $x_{4}\left(0\right)={\left[2.4\;0.0\right]}^{T}$, $x_{5}\left(0\right)={\left[-2.8\;0.0\right]}^{T}$ and $x_{6}\left(0\right)={\left[-2\;0.0\right]}^{T}$.*

**Figure 11:**
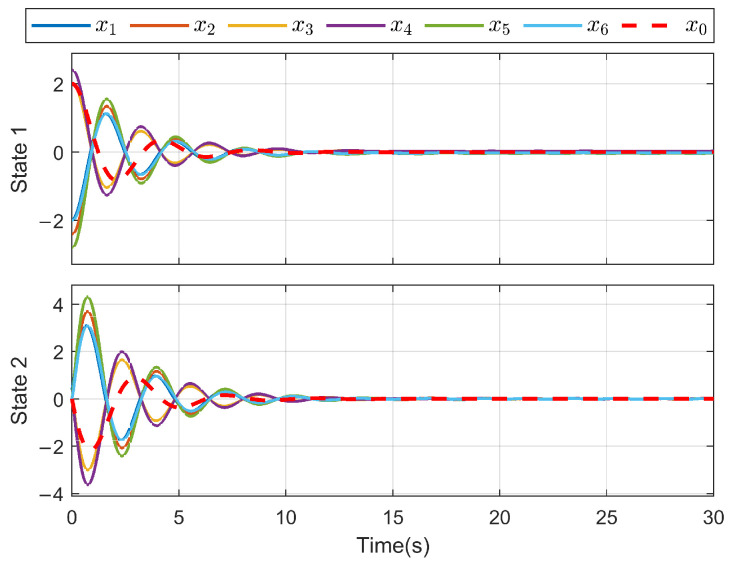
State trajectory for each agent.

**Figure 12:**
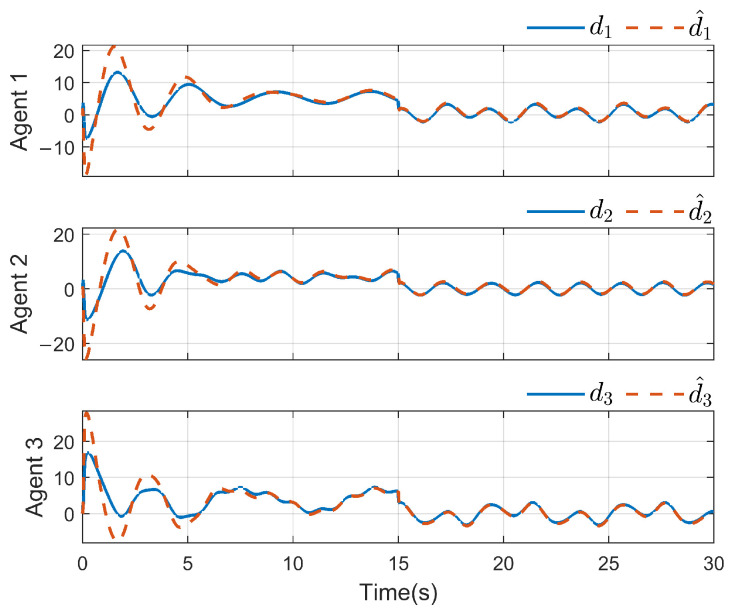
$d_{i}$ and its reconstruction ${\hat{d}}_{i}$(i=1,2,3).

**Figure 13:**
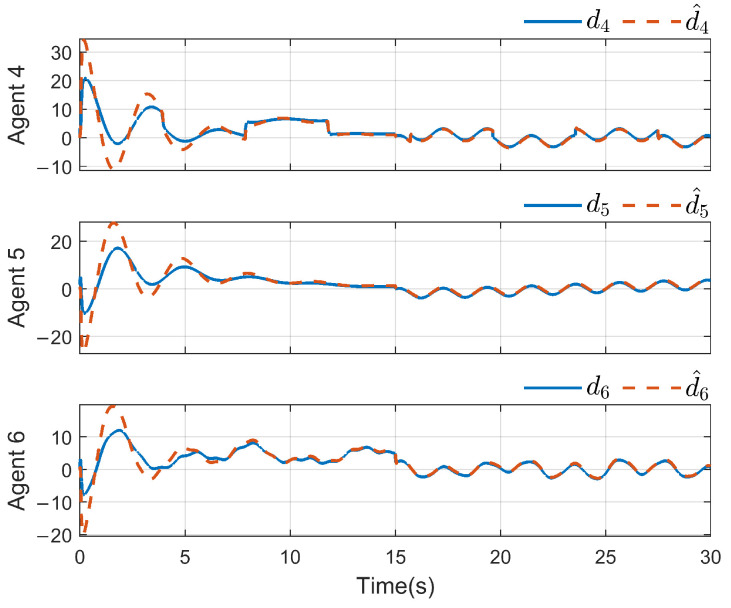
$d_{i}$ and its reconstruction ${\hat{d}}_{i}$(i=4,5,6).

*For the system with directed topology and sparse connections, the states of followers converge to the leader’s state asymptotically as shown in Figure 11. For FR, $Q=\left[\begin{array}{cc} -160.2 & 0\\ 0 & -150.6 \end{array}\right]$ in (13); multiple unknown input $d_{i}$ and its reconstruction ${\hat{d}}_{i}$ are shown in Figure 12 and Figure 13. Clearly, in the case of time-varying topology transformation, the DO-based fault-tolerant control protocol can achieve consensus control successfully.*

## 6. Conclusions

In this paper, a DO-based fault-tolerant control protocol scheme is developed for MAS consensus purposes, where followers can derive the estimated state of the leader based on the introduction of the DO. In fact, the proposed DO is able to estimate the leader’s state asymptotically. Meanwhile, the followers suffer from actuator faults. Thus, a local UIO is designed for each follower to asymptotically estimate the state by decoupling the fault. Moreover, an FR that can estimate the actual fault asymptotically is proposed via an interval observer. The FR is able to decouple the control input of the current follower. Finally, for the *i*th follower agent, using the estimation of the leader’s state provided by the DO (26), the state of the *i*th follower provided by the UIO (9) and the FR (23), a fault-tolerant controller (30) is constructed. Accordingly, a DO-based fault-tolerant control protocol is developed. The DO-based fault-tolerant control protocol is a distributed one, and the distributed feature is majorly reflected by the DO, while the controller is in a centralized form. Further work should focus on DO-based fault-tolerant control protocol construction for heterogeneous and complex formation control tasks.

## Figures and Tables

**Table 2:** Common fault types.

Fault Mode	$\underline{\eta }$	$\bar{\eta }$	$f_{a,i}$
Normal	1	1	$0_{m}$
Outage	0	0	$0_{m}$
Bias	1	1	$\neq 0_{m}$
Stuck	0	0	$\neq 0_{m}$
Loss performance	>0	<1	$0_{m}$

## Data Availability

The original contributions presented in this study are included in the article. Further inquiries can be directed to the corresponding author.

## Abbreviations

The following abbreviations are used in this manuscript:

LMI	Linear Matrix Inequality
UIO	Unknown Input Observer
DIO	Distributed Interval Observer
FR	Fault Reconstruction

## Appendix A. Proof of Lemma 1

According to the condition in Assumption 1, we have

$$\begin{array}{cc} n+m & =\mathrm{rank}\left[\begin{array}{cc} sI_{n}-A & B\\ C & 0 \end{array}\right]\\ \; & =\mathrm{rank}\left(\left[\begin{array}{cc} sI_{n}-A & B\\ C & 0 \end{array}\right]\left[\begin{array}{cc} I_{n} & 0\\ {\left(CB\right)}^{†}CA & I_{m} \end{array}\right]\right)\\ \; & =\mathrm{rank}\left[\begin{array}{cc} sI_{n}-A+B{\left(CB\right)}^{†}CA & B\\ C & 0 \end{array}\right]\\ \; & =\mathrm{rank}\left[\begin{array}{c} sI_{n}-\overset{⌢}{A}\\ C \end{array}\right]+m \end{array}$$

which gives $\mathrm{rank}\left[\begin{array}{c} sI_{n}-\overset{⌢}{A}\\ C \end{array}\right]=n$. Therefore, the matrix pair $\left(\overset{⌢}{A},C\right)$ is detectable.

## Appendix B. Proof of Lemma 4

For Equation (14), ${\bar{\varepsilon }}_{i}\geq 0$ and ${\underline{\varepsilon }}_{i}\geq 0$ hold; the error system (14) needs to be a positive system. By Lemma 2, we have

$${\left(CB\right)}^{+}{\bar{d}}_{i}-{\left(CB\right)}^{-}{\underline{d}}_{i}-CBd_{i}\geq 0$$

$$CBd_{i}-{\left(C_{i}B\right)}^{+}{\underline{d}}_{i}+{\left(CB\right)}^{-}{\bar{d}}_{i}\geq 0$$

holding for all t≥0. Therefore, there exists $\tau_{0}>0$, such that

$$-CA{\tilde{x}}_{i}+{\left(CB\right)}^{+}{\bar{d}}_{i}-{\left(C_{i}B\right)}^{-}{\underline{d}}_{i}-CBd_{i}\geq 0$$

$$CA{\tilde{x}}_{i}+CBd_{i}-{\left(CB\right)}^{+}{\underline{d}}_{i}+{\left(CB\right)}^{-}{\bar{d}}_{i}\geq 0$$

hold while $t\geq \tau_{0}$. By Lemma 2, ${\bar{\varepsilon }}_{i}\left(0\right)\geq 0$ and ${\underline{\varepsilon }}_{i}\left(0\right)\geq 0$. Then, by Lemma 3, *Q* is a Metzler matrix; we conclude that Equation (14) becomes a positive system, and ${\underline{y}}_{i}\left(t\right)\leq y_{i}\left(t\right)\leq {\bar{y}}_{i}\left(t\right)$ holds for all $t\geq \tau_{0}$.
